# Physics-informed deep learning for robust trajectory prediction in automated driving

**DOI:** 10.1038/s41598-026-66767-9

**Published:** 2026-08-17

**Authors:** Anne Stockem Novo

**Affiliations:** Institute of Mechanical Engineering, Westphalian University of Applied Sciences, Gelsenkirchen, Germany

**Keywords:** Engineering, Mathematics and computing, Physics

## Abstract

Planning a safe, comfortable, and energy-efficient ego-vehicle trajectory is fundamental for automated driving. Efficiency reduces emissions, while safety protects occupants and vulnerable road users. Purely data-driven models, however, struggle with robustness and out-of-distribution scenarios, especially for rare edge cases which are underrepresented in the training data. If the data is furthermore affected by sensor noise, the model performance can suffer drastically. Physics-Informed Neural Networks incorporate expert knowledge in form of physical laws or governing equations, enhancing generalisation and transparency compared to standard data-driven models. Only a few studies have applied such networks to the use case automated driving because of lack of reliable physical models for the complex human-driving patterns. This paper investigates the benefit of injecting a physical model, which describes the motion patterns only to a certain extent. It is demonstrated that it still enables a highly accurate extrapolation to OOD scenarios and yields robust performance even with limited data. The root mean squared error on the predicted trajectories is reduced up to a factor of 10.

## Introduction

The predictive power of modern data-driven models is limited by the quantity and fidelity of the training data. In many high-impact domains, when data are protected by intellectual-property constraints, or when manual annotation is prohibitively expensive, large, clean corpora simply do not exist. Recent work shows that embedding expert knowledge into the learning process can mitigate data scarcity and boost performance even on very small datasets^1^. Knowledge can be introduced in several forms, most prominently as Physics-Informed Neural Networks (PINNs)^2^. The usual approach is to extend the loss function with partial differential equations (PDEs) that are otherwise analytically intractable or would demand costly numerical approximations. Complementary strategies include physics-inspired network architectures (e.g., graph neural networks for molecular design^3^), rule-based constraints^4^, and the generation of synthetic data^5^.

Trajectory prediction is an essential part of automated driving. By forecasting the short-term (*łe 10* s) trajectories of surrounding traffic participants, the ego-vehicle can plan a safe path while markedly reducing planning uncertainty. Unlike long-term traffic-flow or route-planning that span minutes, short-term prediction concentrates on the immediate dynamic interactions that directly influence maneuver selection and collision avoidance. Such trajectories are rarely described by a single, closed-form physical law; the classic equation of motion is often too idealised to capture maneuvers such as lane changes, abrupt braking, or cyclist behavior. Consequently, PINNs have been rarely adopted in this field, and an understanding of when and how idealised physical priors can improve data-driven forecasts remains lacking.

In the past, in the field of automated driving specific scenarios have been modeled with PINNs. In dense moving traffic, the longitudinal velocity component can be described well with car-following models like the Intelligent Driver Model (IDM)^6^. In a PINN, the IDM is integrated as additional regularization term in the loss function used for training the neural network^7–9^, demonstrating faster model convergence and error reduction up to 70 % for selected cases^10^. In a similar scenario, the longitudinal component has been modeled with *constant time-headway policy (CTHP)* in order to learn the unknown parameters of adaptive cruise control systems^11^. PINNs have also been used for trajectory reconstruction, where missing sensor information is reconstructed by assuming physics prior knowledge (e.g. vehicle kinematics or boundary conditions)^12^.

Another prominent way of integrating expert knowledge is data augmentation or the use of simulations and digital twins, which have been investigated for various use cases. Digital twins were proposed as a method to overcome the problem of heterogeneous data modalities and avoiding the approximation of theoretical models^13^. An emerging problem is the model robustness for noisy training data, which has achieved attention in the context of PDEs where noisy features with a high aleatoric uncertainty were recognized as a challenge^14^. For a Navier-Stokes Informed Neural Network, it was shown that the model can perform quite robust still on noisy data^15,16^.

In this paper, an LSTM-based PINN is developed for trajectory prediction in automated driving. It is trained and evaluated on the Argoverse 2 dataset, a publicly available, fully annotated sensor dataset. Special focus is given to the extrapolation capabilities on out-of-distribution data and the training efficiency for small datasets. A variation of the dataset size investigates model robustness which is benchmarked against a purely data-driven baseline.

### Novelty and key contributions

The contributions made in this paper are the following: (a) An LSTM-based PINN is compared against a conventional data-driven model for short-term trajectory prediction. Unlike previous studies, the modelling is not limited to the longitudinal component. (b) Model extrapolation is studied, demonstrating that physics-informed models retain robust for out-of-distribution scenarios even if the physical model describes the real data only to a certain extent. (c) A systematic empirical study is conducted, analyzing how physical knowledge impacts predictive performance, training convergence speed, and robustness when training data is scarce or noisy.

## Theoretical framework and integration of physical knowledge

### Trajectory prediction

Short-term trajectory prediction is a regression problem which aims to forecast the future trajectories of other traffic participants in the time horizon up to *∼ * 10 s. For a given set of recorded time series trajectory coordinates, the first *k* samples are taken as the model input, $z_{-k...0} = (x_{-k+1},y_{-k+1}), ..., (x_0, y_0)$, while the later part of the trajectory is the label, $z_{1...T} = (x_1,y_1), ..., (x_{T}, y_{T})$. The goal is to predict the labeled trajectory with minimum deviation. In order to avoid too large label values and to achieve better training convergence, typically, the coordinate deltas $\Delta z_{i}=(\Delta x_i, \Delta y_i) = (x_{i+1} - x_{i}, y_{i+1} - y_{i})$ are modelled instead of the absolute coordinate values.

More formally, let $\textbf{X}$ be the input data to a data-driven model, *z* be the expected model output or label, and $\hat{z} = f(\textbf{X}| \theta )$ the actual model output or prediction with *f* a non-linear transfer function and model parameters *θ *. The aim is to find the set of optimal model parameters which minimize a model objective function.

For regression problems, such as trajectory prediction, the objective or loss function *L* for finding the optimal set of model parameters is typically the *mean squared error* (MSE). To compute the loss, the predicted output $\hat{z}= (\hat{x}, \hat{y})$ is compared against the expected output *z = (x, y)* and accumulated for all data in the training set. The loss is finally averaged over all training samples *N,*

1 $$\begin{aligned} L(\textbf{X}| \theta ) = \frac{1}{2N} \sum _{n=1}^N \left[ \left( x_n - \hat{x} _n \right) ^2 +\left( y_n - \hat{y} _n \right) ^2 \right] . \end{aligned}$$

In order to find the best model parameters $\theta ^* = \min _{\theta } L(\textbf{X}| \theta )$, it is optimized with Stochastic Gradient Descent.

### Physical model

A constant velocity model is chosen as the physical model. This simple and deliberately imperfect physical motion model is chosen in order to be able to trace back the determining factors. This makes it possible to isolate the effects on model performance that originate either from real physics or from idealized data.

**Figure 1:**
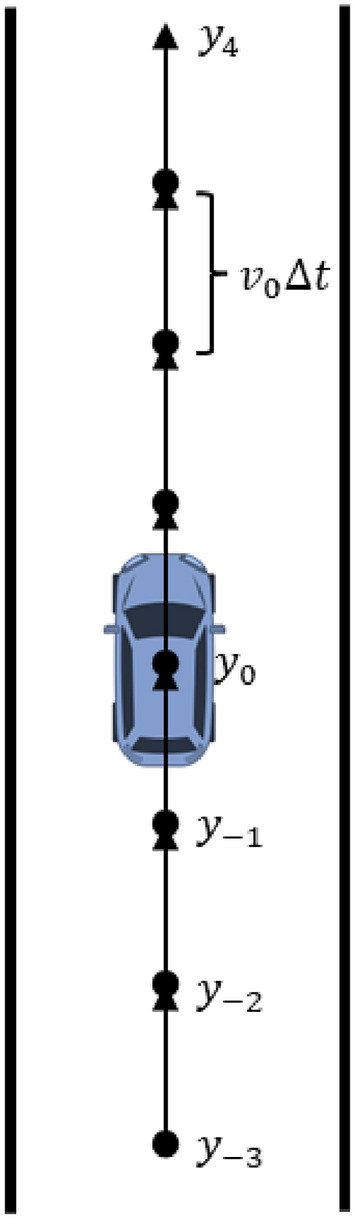
Constant velocity model for trajectory prediction with $\Delta t = t_{i+1} - t_i$.

The constant velocity model is described by *d z / dt = const* for the two-dimensional spatial coordinate *z(t)= (x(t),y(t))* and time *t*. Without loss of generality, a coordinate transformation can be applied such that the direction of travel is aligned with the *y* axis as shown in Fig. 1.

The equation of motion is thus given by

2 $$\begin{aligned} z_{phys}(t) = y_0 + v_0 \, t, \end{aligned}$$

where $v_0$ is a constant one-dimensional vector which can be determined from the trajectory history as

3 $$\begin{aligned} v_0 = \frac{y_0 - y_{-s}}{s \Delta t}, \end{aligned}$$

where *s* is the number of lookback steps.

This physical model can be integrated into the neural network model. So-called *collocation data* is generated with the constant velocity model, Eq. (2), and handed to the neural network during training. It serves as an additional regularization term during the learning process, such as

4 $$\begin{aligned} L_{PINN} = L_{data} + L_{phys}, \end{aligned}$$

where $L_{data}$ is the mean squared error loss from Equation (1) and $L_{phys}$ is the mean squared error loss from the constant velocity model. The loss can then be written as

5 $$\begin{aligned} L_{PINN} =\frac{1}{2N_o} \sum _{n=1}^{N_o} \sum _{i=1}^{2} \left( z_{i,n} - \hat{z}_{i,n} \right) ^2 + \frac{1}{2N_c} \sum _{n=1}^{N_c} \sum _{i=1}^{2} \left( z_{phys, {i,n}} - \hat{z}_{i,n} \right) ^2 \end{aligned}$$

with $N_o$ the number of real observation data samples, $N_c$ the number of synthetic collocation data samples, and $z_{phys,n}$ the solution of Eq. (2). Different weighting schemes between data loss and physical loss have been tested, and it was found that equal weighting consistently yielded robust performance.

## Experiments

### Data

The real sensor data is a subset of the publicly available *Argoverse 2 Motion Forecasting Dataset*^17,18^. *Argoverse2* is a collection of autonomous driving data and high-definition maps from six U.S. American cities recorded with a multi-modal synchronized sensor setup: two 64-beam Lidars with a view range of 200 m, seven ring cameras providing $360^\circ$ view, two front-view stereo cameras and GPS localization which is combined with sensor localization for 6-DOF. The *Motion Forecasting Dataset* consists of 250,000 scenarios for training and validation, each 11 s long (5 s observation/6 s prediction) with a 10 Hz sample rate. The agents have been tracked and labelled with ten unique object classes. Each scenario follows one focal agent in close distance ahead of the measurement vehicle for which the complete track over 11 s is available.

**Figure 2:**
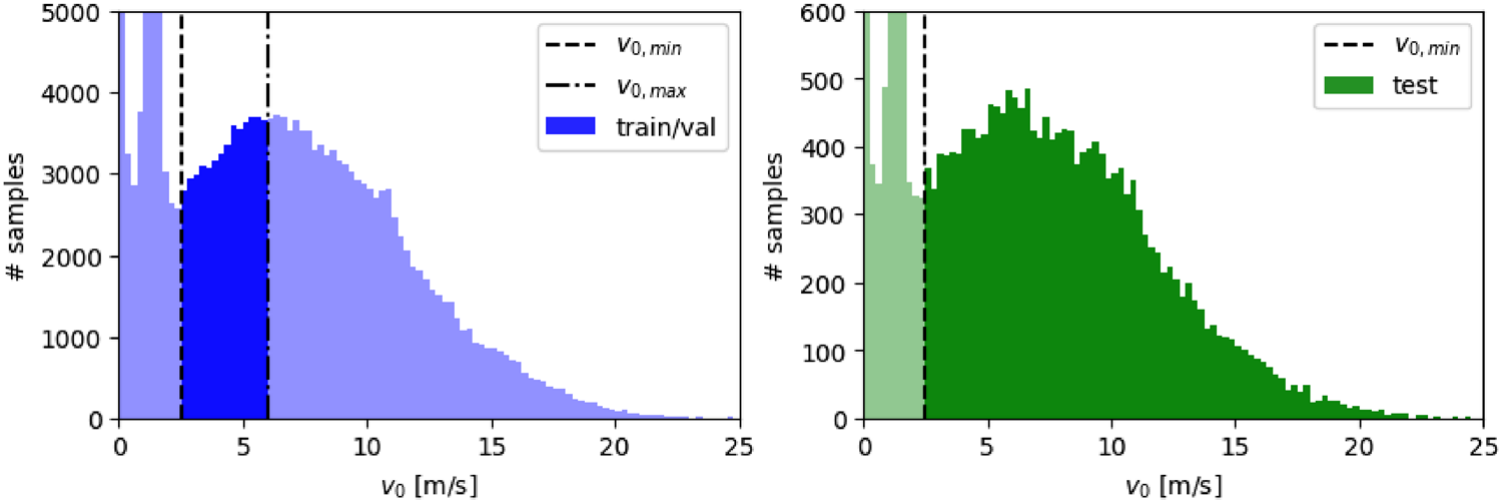
Velocity histograms at observation time for training/validation data (left) and test data (right). The dashed lines indicate the filter thresholds.

The distribution of velocities at observation time in the Argoverse 2 dataset is shown in Fig. 2 for training/validation (left) and test data (right). Two separate distributions can be identified, the first with a peak at very small velocities ($v_0 < 2.5$ m/s), the second for $v_0 > 2.5$ m/s with a long tail up to velocities of *∼ * 30 m/s. The first distribution covers typical urban scenarios like stop-and-go, searching for parking spots, or turning manoeuvres at very low speeds. Since this is not the focus of this study and forward moving traffic is required, the dataset is filtered for motorized vehicles with driving speeds $v_0 \ge 2.5$ m/s.

The training data is further limited to a maximum driving speed of 6 m/s in order to test the extrapolation capabilities of the model. This leaves 46,423 out of 199,908 training scenes (23,2 %) and 18,083 out of 24,988 test scenes (72,4 %). The training data is split into 80:20 ratio of training to validation data. All scenarios are aligned with the positive y-axis and translated to position the focal agent at observation time $$t_0 = 5 $$ s at spatial coordinates $$(x_0,y_0) = (0,0)$$.

To study the impact of the PINN modelling, the data is analysed with respect to their deviation from a constant velocity model. The deviation criterion is the MSE along the labeled future 6 s trajectory for each scenario,

6 $$\begin{aligned} CV MSE_n = \frac{1}{T} \sum _{t=1}^T \left| \left| z_{n,t} - z_{n, t,phys} \right| \right| ^2, \end{aligned}$$

with prediction time points $t = \{1, 2, 3, 4, 5, 6\} s$.

**Figure 3:**
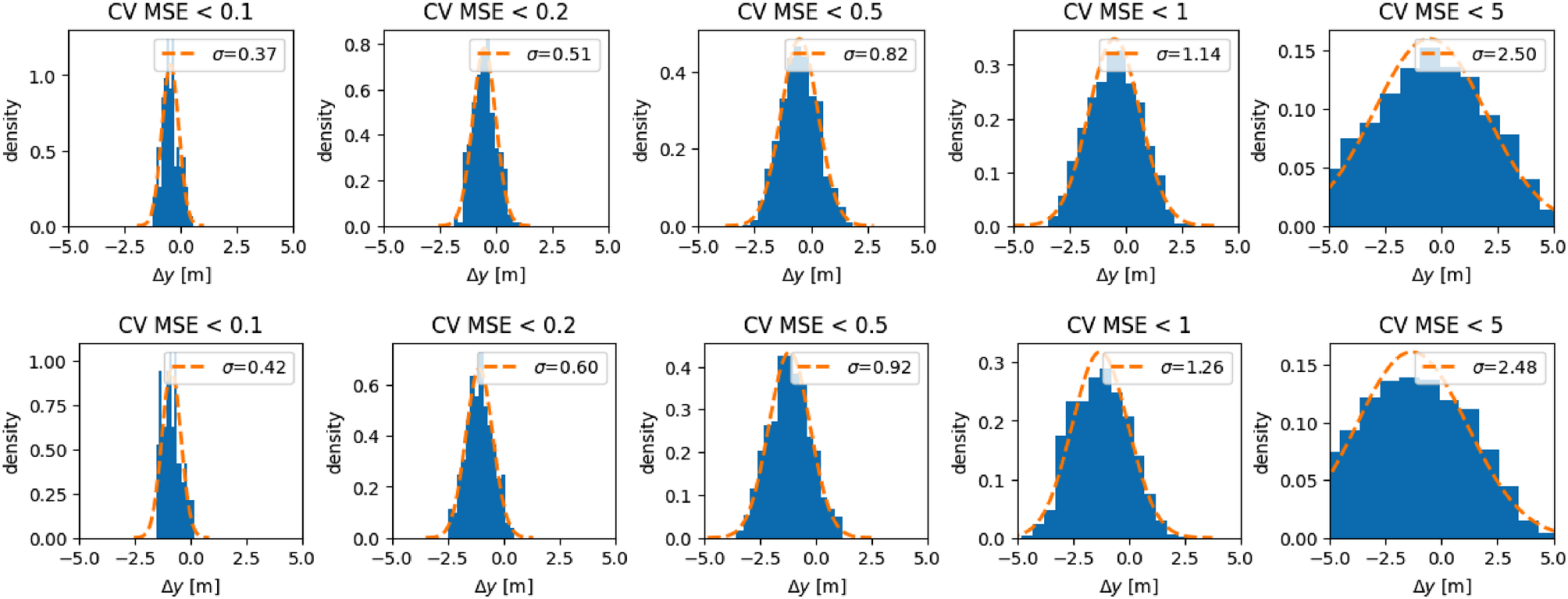
Distributions of the deviation from a constant velocity model, *Δ y* at final prediction time point *T=6* s for the different *CV MSE* ranges. The dashed lines show a Gaussian fit to the data. Top: train data, bottom: test data.

In the experiments, the model performance is investigated for data within defined CV MSE ranges, with threshold values $CV MSE =\{0, 0.1, 0.2, 0.5, 1, 5\}$m$^2$. The distribution of the deviation at final prediction time point *T=6* s over all scenarios within each *CV MSE* range is shown in Figure 3.

They can be fitted well with Gaussian distributions with corresponding variances $\sigma [m] = \{ 0.37, 0.51, 0.82, 1.14, 2.50\}$. A slight shift towards negative mean values is observed, meaning that more trajectories deviate towards deceleration than acceleration. For the following experiments, the data is filtered with respect to their *CV MSE* values. Table 1 lists the parameters used in the experiments. The input history is limited to 2 s with a sample frequency of 2 Hz. The trajectory labels are sampled with 1 Hz, such that the prediction time points are $t =\{1, 2, 3, 4, 5, 6\}$ s. The sampling frequency was lowered in order to reduce the number of data points, which speeds up computation and helps preventing overfitting, since this reduced rate still captures the necessary information.

**Table 1:** Parameter specification for the training and test datasets.

CV MSE [m]	* < 0.1 *	* < 0.2 *	* < 0.5 *	* < 1 *	* < 5 *
*#* train samples	128	324	909	1722	5609
$v_{0} \le 6$ m/s	128	196	585	813	3887
*#* test samples	82	263	767	1581	4512
LSTM units	32	32	128	128	128
Batch size	4	4	8	16	16

### Model and training

**Figure 4:**
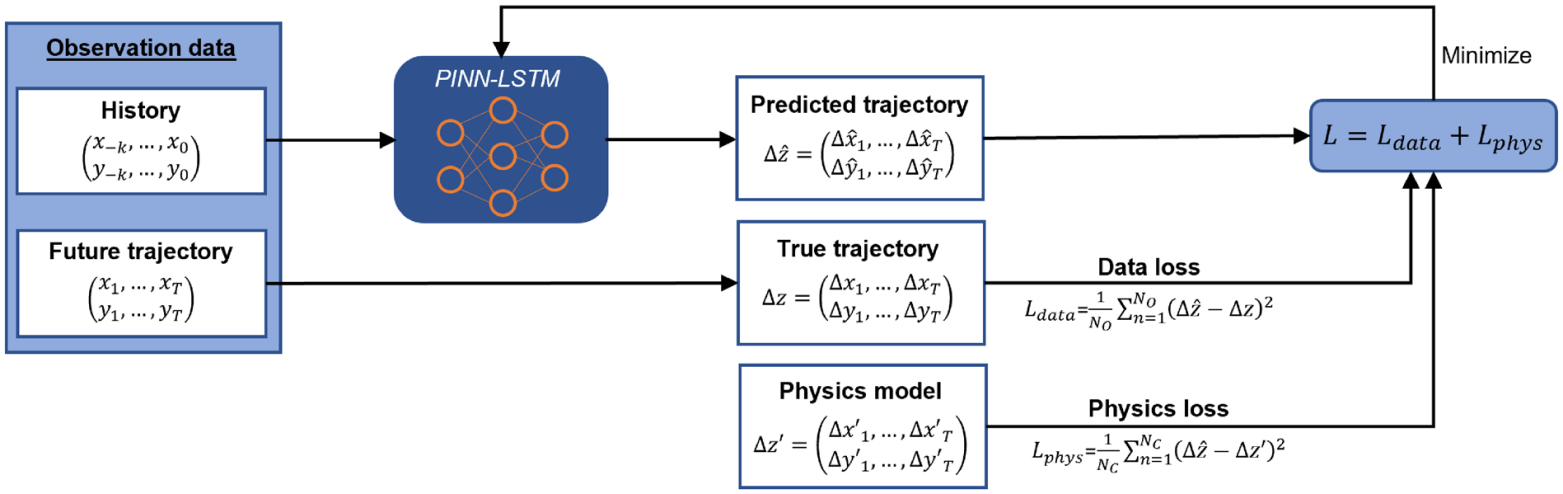
The data flow in the physics-informed neural network.

The trajectory modelling is done with a one-layered long-short term memory (LSTM) network. The number of parallel LSTM units is varied depending on the number of samples in the dataset and specified in Table 1. Figure 4 shows the data flow in the physics-informed neural network.

The reference model is optimized on the data loss only, and serves as a benchmark for the PINN model. The training is done without limiting the train data to a maximum velocity, as well as with limiting it to $$v_0 \in [2.5, 6] $$ m/s. Four different experimental settings are compared: the reference model which is trained with data covering the full spectrum of velocities $$v_0$$ (ref), the reference model with limiting the training data to $$v_0 = 6 $$ m/s (ref-vmax), the physics-informed neural network (pinn) and the physics-informed neural network with limiting the training data to $$v_0 = 6 $$ m/s (pinn-vmax). The model hyper-parameters are optimized and the best epoch is selected with early stopping on the validation data. The number of physics samples, which contribute to the physics loss as shown in Fig. 4, is 4000.

The evaluation metric for quantifying the model performance is the *root mean squared error*,

7 $$\begin{aligned} RMSE = \sqrt{\frac{1}{M} \sum _{m=1}^{M} \left[ \left( x_m - \hat{x}_m \right) ^2 + \left( y_m - \hat{y}_m \right) ^2 \right] }, \end{aligned}$$

where *M* is the number of samples under test.

## Results and discussions

### Benchmark and PINN comparison

**Figure 5:**
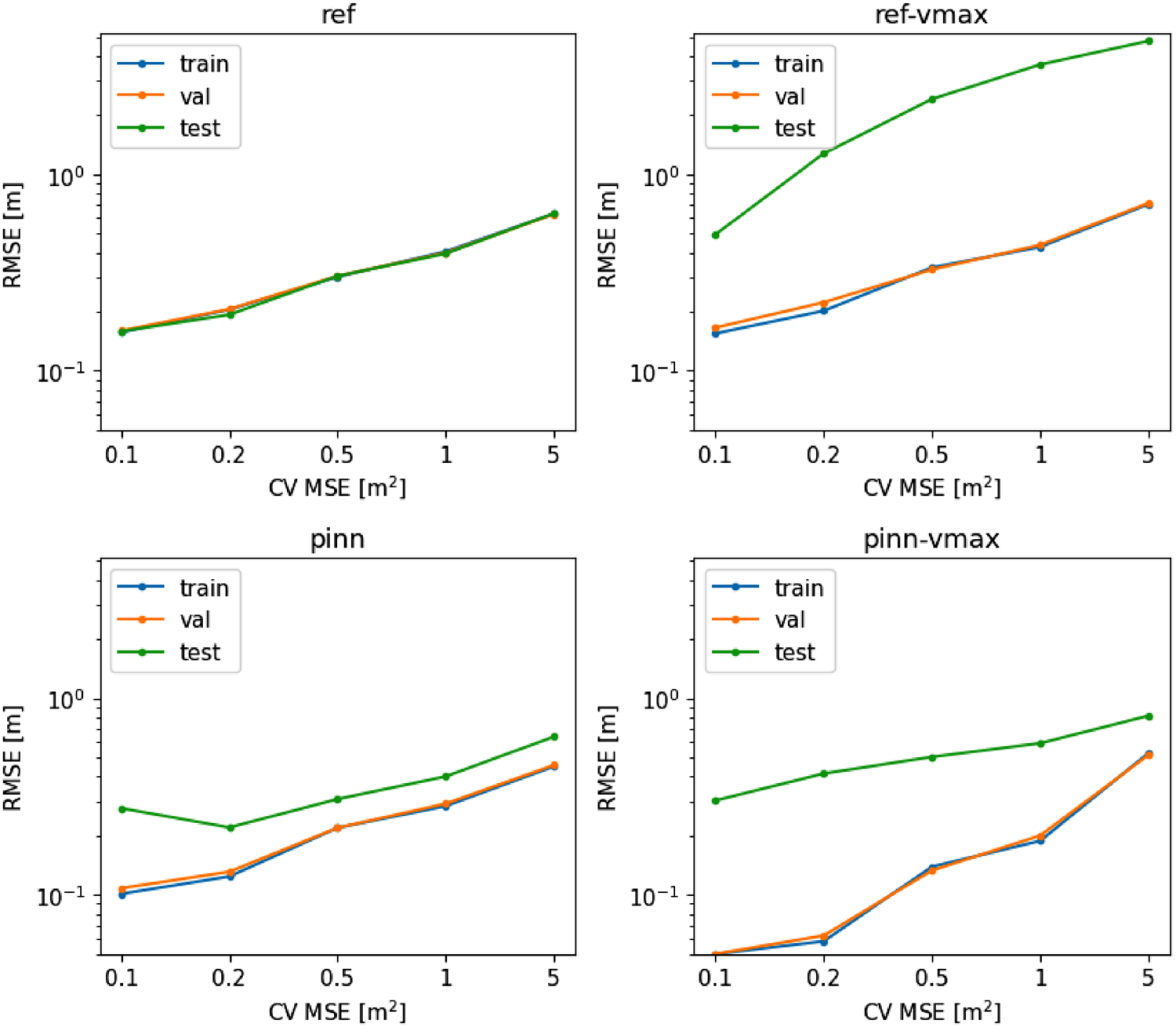
RMSE for training, validation and test data versus *CV MSE* (top left: training on real data (ref), top right: training on reduced real data ($v_0 \le 6$ m/s) (ref-vmax), bottom left: training on real data and synthetic data (pinn), bottom right: training on reduced real data and synthetic data (pinn-vmax).

At first, the overall performance of the benchmark and the PINN models are compared. Figure 5 shows the model error RMSE on the training, validation and test datasets defined by Eq. (7) versus varying *CV MSE* ranges. The training and validation curves are almost identical for all runs which guarantees that there is no overfitting even for the runs with a very small number of data samples.

For the reference model which had access to the entire training data, all three RMSE curves for training, validation and test data are in perfect agreement (Fig. 5 top left). In contrast, for the reference model which had access to only a limited fraction of the training data (ref-vmax, Fig. 5 top right), the test RMSE is more than a magnitude larger than that of the training and validation data. This result was expected since the data with velocities $v_0 \ge 6$ m/s is out-of-distribution data. On the other hand, the PINN models (pinn and pinn-vmax, Fig. 5 bottom) show an enhanced training and validation RMSE, as well as a test RMSE which is comparable to the test RMSE of the reference model (ref). The detailled test RMSE values are reported in Table 2.

**Table 2:** Test RMSE values for the different models.

	Test RMSE [m]			
CV MSE [m$^2$]	ref	ref-vmax	pinn	pinn-vmax
*[0.0, 0.1]*	0.159	0.495	0.276	0.302
*[0.1, 0.2]*	0.193	1.278	0.220	0.414
*[0.2, 0.5]*	0.304	2.417	0.307	0.503
*[0.5, 1.0]*	0.395	3.625	0.400	0.591
*[1.0, 5.0]*	0.633	4.774	0.638	0.815

The results are further investigated in more detail in order to better understand the differences of the PINN model towards the reference model in terms of extrapolation capabilities, model efficiency and robustness.

### Exrapolation

**Figure 6:**
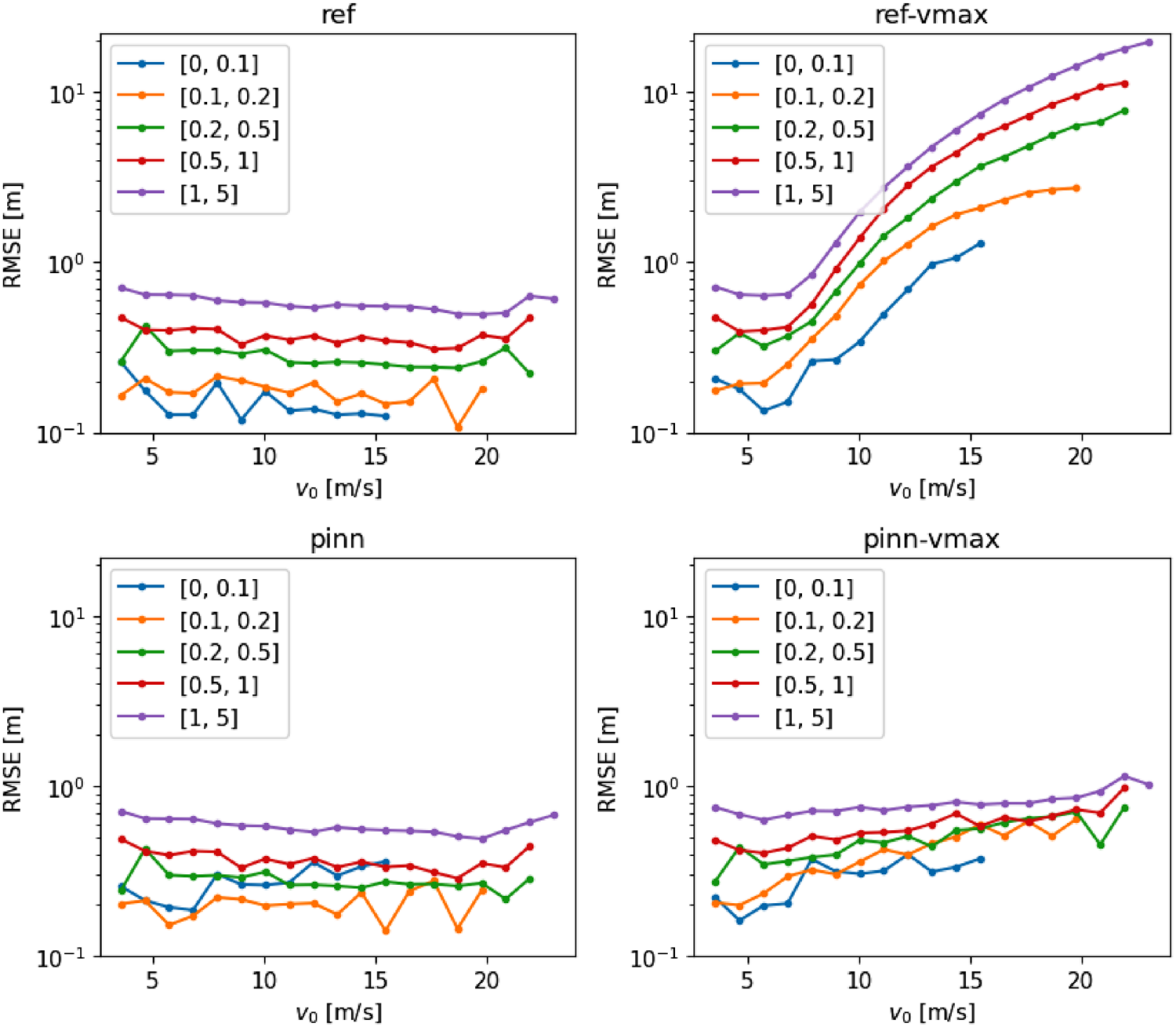
RMSE for test data versus velocity at observation time $v_0$ for varying CV MSE (top left: training on real data (ref), top right: training on reduced real data ($v_0 \le 6$ m/s) (ref-vmax), bottom left: training on real data and synthetic data (pinn), bottom right: training on reduced real data and synthetic data (pinn-vmax).

In order to study extrapolation beyond the in-distribution training data, the training and validation data was limited to $v_0\in [2.5, 6]$ m/s, while the test data covers the full range of velocities $v_0 \ge 2.5$ m/s. The velocity range *[2.5, 6]* m/s has been split into 19 equal distant bins and the RMSE values have been averaged over all scenarios within each bin. Figure 6 shows these RMSE values on the test dataset for the different $v_0$ intervals.

The reference and the PINN models, which have access to the entire training dataset, retain a relatively stable test RMSE over $$v_0$$, which is shown in the left column of Fig. 6. The models with access to only the reduced dataset (right column of Fig. 6) show the same flat test RMSE, independent of $$v_0$$, only up to $$v_0 = 6 $$ m/s. The values are almost identical as for the models in the left column. For the ref-vmax model, for velocities $$v_0 > 6 $$ m/s the test RMSE monotonously increases, reaching values nearly two magnitudes larger than the reference model. The PINN model (pinn-vmax) exhibits a flat curve similar to the reference model, showing very weak dependence on $$v_0$$ over the whole velocity range (Fig. 6 bttom right). It can be concluded that the reference and pinn models are robust to changes in $$v_0$$, while the reference model trained on a reduced-dataset performs well only within the velocity range it has seen during training. In contrast, the PINN model (pinn-vmax) maintains stable accuracy even when extrapolating to higher velocities.

**Figure 7:**
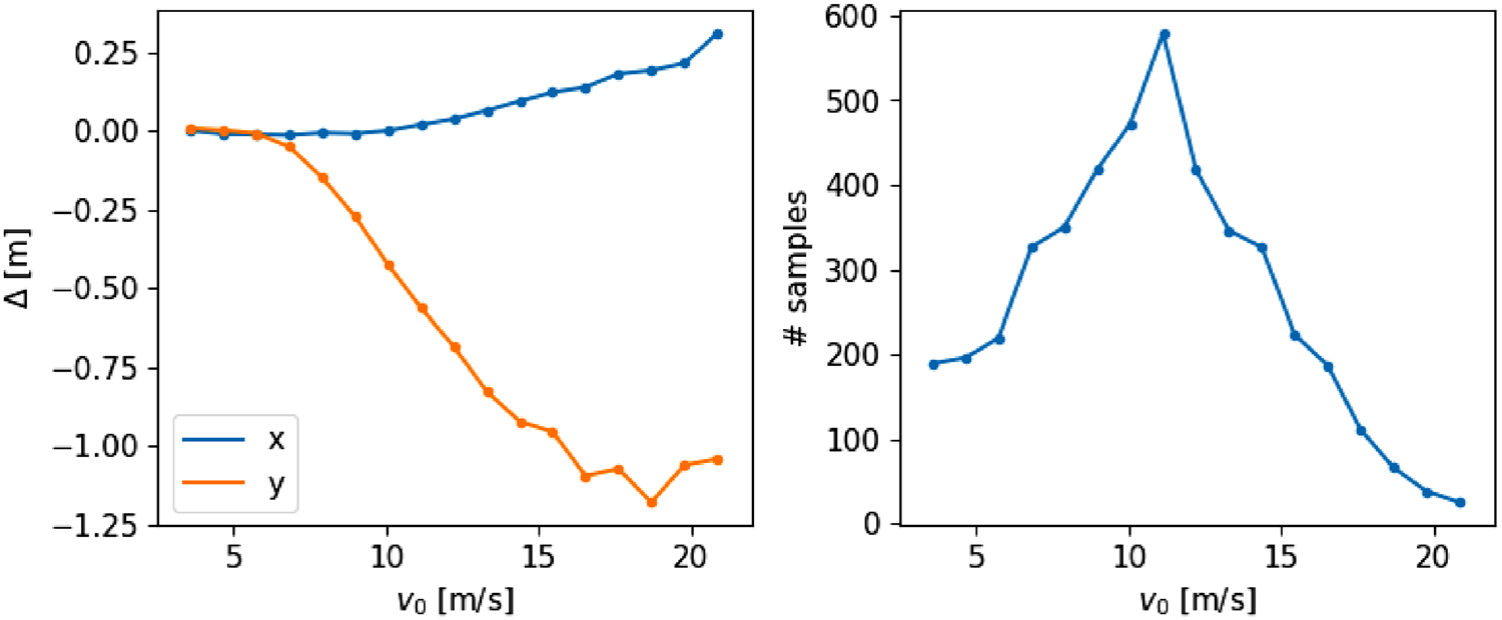
Left: Average differences between predicted and labelled *x* and *y* coordinates for test data versus velocity at observation time $v_0$, shown for the ref-vmax model. Right: Number of samples in a corresponding velocity bin in the test dataset. The saturation at large velocities is not meaningful since the number of test data samples is extremely low.

The gradual rise of the test RMSE for the ref-vmax model is noteworthy, because all samples with $$v_0 > 6 $$ m/s are out-of-distribution and any model result would be reasonable. This behavior can be understood by recognizing that higher initial velocities generate longer driven paths. The model tends to underestimate the trajectory length which can be confirmed by the delta differences between predicted and annotated *x* and *y* coordinates shown in Fig. 7. Therefore, the discrepancy between prediction and ground truth grows with the path length. The same relative error translates into a larger absolute deviation for long range motions. Consequently, the accumulated error increases smoothly as $$v_0$$ exceeds the velocity range seen during training.

### Training convergence

The modelling efficiency was studied by analyzing the number of epochs required until model convergence.

**Figure 8:**
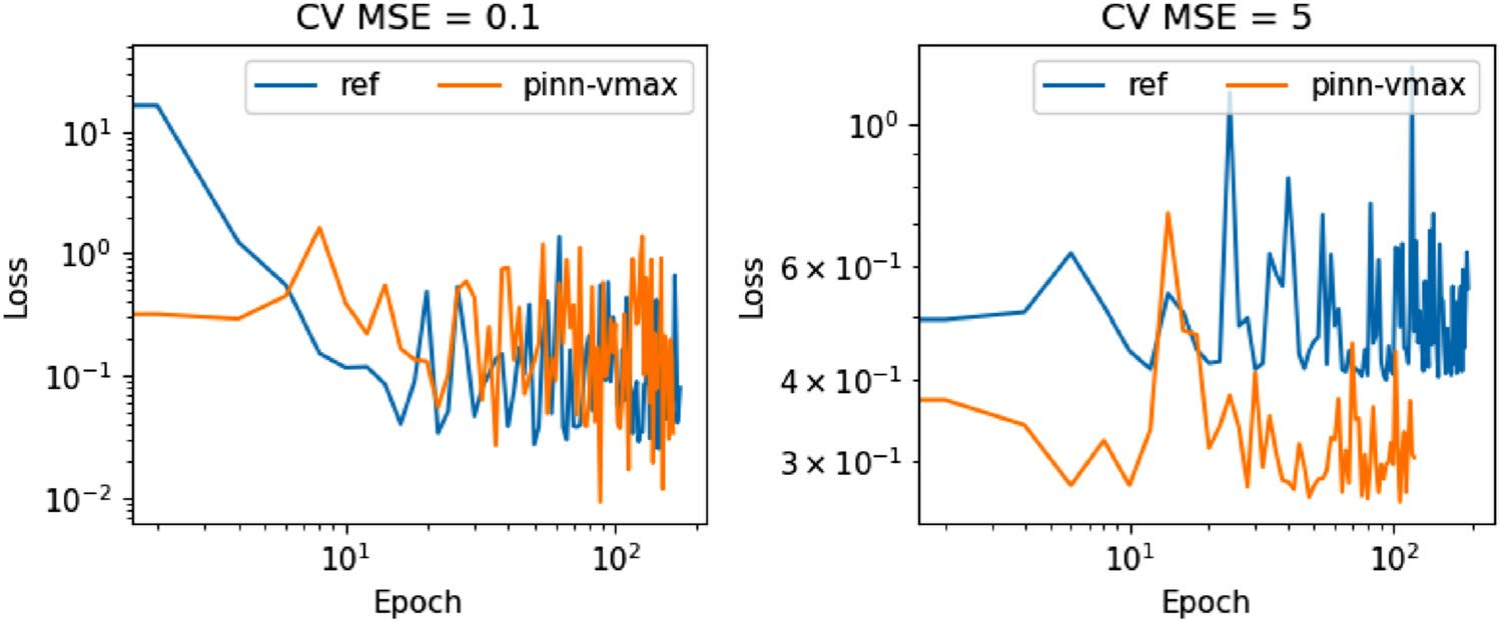
Validation loss versus training epoch for the different models and varying values of *CV MSE*.

Figure 8 shows the validation loss per epoch for the lowest and highest *CV MSE* values. For *CV MSE = 0.1*, the reference model has a slower initial learning curve with a sharp decrease after *∼ 100* epochs which is not observed for *CV MSE = 5*. For the PINN model, the final loss on the validation data is not as good as for the reference model, in agreement with the previous results shown in Fig. 5.

For *CV MSE=5*, the PINN model has a lower validation loss, reduced by a factor of 2 compared to the reference model. Convergence is reached very quickly. The loss finally oscillates around a minimum value, which is caused by the competing terms of data and physics loss.

To conclude, the PINN model reaches convergence slightly earlier and is therefore more efficient in training than the reference model.

### Model robustness

The experimental results presented in the previous subsections are analysed more deeply by varying the number of samples for a fixed *CV MSE* range.

**Figure 9:**
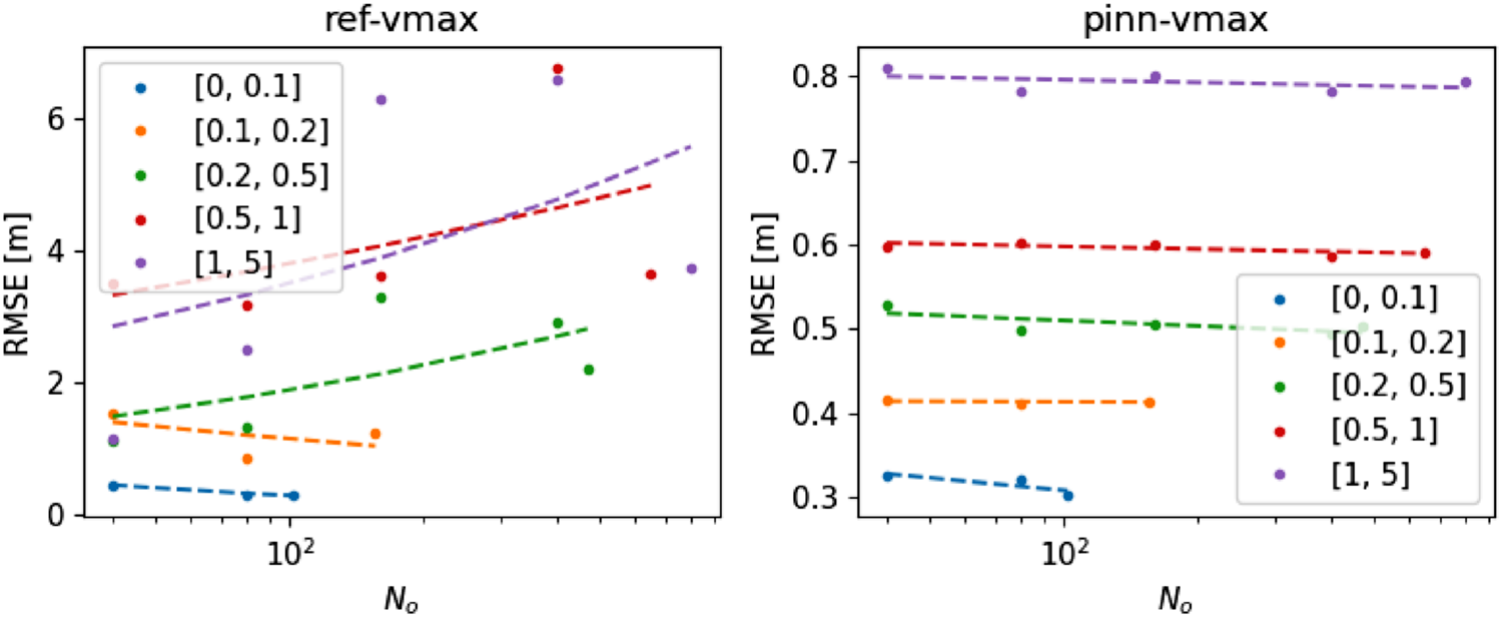
Test RMSE versus number of real training samples $N_o$ for the different models (left: training on reduced real data ($v_0 \le 6$ m/s) (ref-vmax), right: training on reduced real data and synthetic data (pinn-vmax)). The dashed lines are power law fits from Eq. (8) to the data.

Figure 9 compares the test RMSE versus varying number of real training samples $N_o$. Power law distributions have been fitted to the data, described by

8 $$\begin{aligned} f(N_o) = a N_o^{-b}, \end{aligned}$$

where *a* is a scaling factor and *b* is the power. The determined parameter values are listed in Table 3.

**Table 3:** Empirically determined parameters from power law Eq. (8).

	ref-vmax		pinn-vmax	
CV MSE [m$^2$]	*a*	*b*	*a*	*b*
*[0.0, 0.1]*	2.7	0.49	0.4	0.07
*[0.1, 0.2]*	3.1	0.22	0.4	0.00
*[0.2, 0.5]*	0.6	0.26	0.6	0.02
*[0.5, 1.0]*	1.9	0.15	0.6	0.01
*[1.0, 5.0]*	1.2	0.22	0.8	0.01

The reference model ref-vmax shows the expected decrease of the test RMSE with number of samples only for small *CV MSE* values, which are trajectories that can be also described well by a constant velocity model. For larger values, the distribution of trajectories gets too wide such that the number of sampled trajectories is not sufficient to generalize a trajectory pattern. Moreover, and more importantly, the missing representations of larger velocities, $v_0 > 6$ m/s dominates for larger *CV MSE* values. As a result, the model prediction is not meaningful at all and almost random. This breaking in behavior is observed for *CV MSE > 0.2*, which is correlated with the test RMSE value, since $\sqrt{CV MSE} > 0.45$ is comparable to the test RMSE *≲ 1*.

Contrary, the PINN model (pinn-vmax) shows the expected increasing model performance (= reduced RMSE) with larger number of training samples. Furthermore, a much weaker scaling is observed with a power *b ≪ 1 *, which is due to the larger number of collocation samples used for regularization.

It can be concluded that the data-driven model sufficiently benefits from the physical knowledge injection. Although the model performance decreases if the collocation data deviates from the real data, an enormous performance improvement and stabilization of model robustness is observed.

## Conclusions

Planning a safe, comfortable, and energy-efficient trajectory is essential for automated driving. However, data-driven models are sensitive to limited training data, sensor noise, and out-of-distribution (OOD) scenarios or rarely occurring edge cases. Such deficiencies can thus lead to a drastic performance degradation. By embedding a constant velocity physical model within an LSTM-based neural network, the proposed Physics-Informed Neural Network (PINN) leverages expert knowledge to regularize learning and compensate for the scarcity of representative samples. Experiments on the publicly available Argoverse 2 dataset demonstrate that this simple physical prior improves short-term trajectory prediction. The root mean squared error (RMSE) is reduced by up to a factor of ten, the model converges slightly earlier than the purely data-driven baseline, and its performance remains stable even when extrapolating to velocities far beyond those seen during training.

The empirical study further reveals that the robustness of the PINN is grounded in its ability to retain meaningful predictions despite a mismatch between the injected physics and the true vehicle patterns. While the reduced-dataset reference model collapses rapidly once the initial velocity exceeds the training range, the PINN continues to deliver accurate forecasts, indicating that the physical loss acts as an effective regularizer that mitigates overfitting to noisy or incomplete data. This behavior confirms that even an imperfect physical description can substantially enhance generalisation and transparency. PINNs can therefore be identified as promising for addressing the reliability gaps that currently limit the deployment of data-driven trajectory predictors in safety-critical autonomous-driving systems.

Future work will address extending the framework with more realistic physical models, such as car-following models or multi-modal maneuver representations. More complex neural network architectures will be explored, as well as real-time implementation on embedded automotive hardware. The trade-off between data loss and physics loss has been investigated empirically for a limited number of weight values and model settings. A comprehensive analysis of how the weighting influences model performance is planned for a forthcoming study. Furthermore, the out-of-distribution extrapolation capability of PINNs will be explored based on the scenario type (lane keeping, lane changing, turning etc.).

## Data Availability

The data can be made available on request.

## References

[1] Lucor, D., Agrawal, A. & Sergent, A. Simple computational strategies for more effective physics-informed neural networks modeling of turbulent natural convection. *J. Comput. Phys.* **456**, 111022 (2022).

[2] von Rueden, L., Garcke, J. & Bauckhage, C. How does knowledge injection help in informed machine learning? In *2023 International Joint Conference on Neural Networks (IJCNN)*, 1–8, 10.1109/IJCNN54540.2023.10191994 (2023).

[3] Atz, K., Grisoni, F. & Schneider, G. Geometric deep learning on molecular representations. *Nat. Mach. Intell.* **3**, 1023–1032 (2021).

[4] Abrishami, S., Khakzad, N. & Hosseini, S. M. A data-based comparison of bn-hra models in assessing human error probability: An offshore evacuation case study. *Reliability Eng. Syst. Safety* **202**, 107043 (2020).

[5] Zapf, B. et al. Investigating molecular transport in the human brain from mri with physics-informed neural networks. *Sci. Rep.* **12**, 15475 (2022). 36104360 10.1038/s41598-022-19157-w PMC9474534

[6] Treiber, M. & Helbing, D. Explanation of observed features of self-organization in traffic flow. *arXiv preprint *arXiv:cond-mat/9901239 (1999).

[7] Mo, Z., Shi, R. & Di, X. A physics-informed deep learning paradigm for car-following models. *Transport. Res. Part C Emerg. Technol.* **130**, 103240 (2021).

[8] Ma, L., Qu, S., Song, L., Zhang, Z. & Ren, J. A physics-informed generative car-following model for connected autonomous vehicles. *Entropy* **25** (2023). 10.3390/e25071050 PMC10378484 37509998

[9] Tischmann, P., Baumann, R. & Novo, A. S. Physics informed deep learning for motion prediction in autonomous driving. In *AmEC 2024–Automotive meets Electronics & Control; 14. GMM Symposium*, 7–12 (VDE, 2024).

[10] Geng, M., Li, J., Xia, Y. & Chen, X. M. A physics-informed transformer model for vehicle trajectory prediction on highways. *Transport. Res. Part C Emerg. Technol.* **154**, 104272. 10.1016/j.trc.2023.104272 (2023).

[11] Apostolakis, T. & Ampountolas, K. Physics-inspired neural networks for parameter learning of adaptive cruise control systems. *IEEE Transactions on Vehicular Technology* (2024).

[12] Long, K., Shi, X. & Li, X. Physics-informed neural network for cross-dynamics vehicle trajectory stitching. *Transport. Res. Part E Logistics Transport. Rev.* **192**, 103799. 10.1016/j.tre.2024.103799 (2024).

[13] Görtz, M. Digital twins: Past, present and future. *Sci. Rep.* **16**, 10510 (2026). 41896657 10.1038/s41598-026-45272-z PMC13031716

[14] Karniadakis, G. E. et al. Physics-informed machine learning. *Nat. Rev. Phys.* **3**, 422–440 (2021).

[15] Raissi, M., Yazdani, A. & Karniadakis, G. E. Hidden fluid mechanics: Learning velocity and pressure fields from flow visualizations. *Science* **367**, 1026–1030. 10.1126/science.aaw4741 (2020). 32001523 10.1126/science.aaw4741 PMC7219083

[16] Yang, L., Meng, X. & Karniadakis, G. E. B-pinns: Bayesian physics-informed neural networks for forward and inverse pde problems with noisy data. *J. Comput. Phys.* **425**, 109913. 10.1016/j.jcp.2020.109913 (2021).

[17] Wilson, B. Argoverse 2: Next generation datasets for self-driving perception and forecasting. In Proceedings of the Neural Information Processing Systems Track on Datasets and Benchmarks (NeurIPS Datasets and Benchmarks 2021 (2021).

[18] Lambert, J. & Hays, J. Trust, but verify: Cross-modality fusion for hd map change detection. In *Proceedings of the Neural Information Processing Systems Track on Datasets and Benchmarks (NeurIPS Datasets and Benchmarks 2021)* (2021).

